# A Pruning Neural Network Model in Credit Classification Analysis

**DOI:** 10.1155/2018/9390410

**Published:** 2018-02-11

**Authors:** Yajiao Tang, Junkai Ji, Shangce Gao, Hongwei Dai, Yang Yu, Yuki Todo

**Affiliations:** ^1^Faculty of Engineering, University of Toyama, Toyama-shi 930-8555, Japan; ^2^College of Economics, Central South University of Forestry and Technology, Changsha 410004, China; ^3^School of Computer Engineering, Huaihai Institute of Technology, Lianyungang 222005, China; ^4^School of Electrical and Computer Engineering, Kanazawa University, Kanazawa-shi 920-1192, Japan

## Abstract

Nowadays, credit classification models are widely applied because they can help financial decision-makers to handle credit classification issues. Among them, artificial neural networks (ANNs) have been widely accepted as the convincing methods in the credit industry. In this paper, we propose a pruning neural network (PNN) and apply it to solve credit classification problem by adopting the well-known Australian and Japanese credit datasets. The model is inspired by synaptic nonlinearity of a dendritic tree in a biological neural model. And it is trained by an error back-propagation algorithm. The model is capable of realizing a neuronal pruning function by removing the superfluous synapses and useless dendrites and forms a tidy dendritic morphology at the end of learning. Furthermore, we utilize logic circuits (LCs) to simulate the dendritic structures successfully which makes PNN be implemented on the hardware effectively. The statistical results of our experiments have verified that PNN obtains superior performance in comparison with other classical algorithms in terms of accuracy and computational efficiency.

## 1. Introduction

In the past few decades, credit classification has been continuously attracting a great deal of attention from academic researchers and financial institutions, resulting in various algorithms, known as the credit classification models [1]. Credit classification fits for predicting potential risk corresponding to credit portfolio; thus, it plays a fundamental role for financial institutions to improve their liquidity and reduce any possible risk [2]. Concerning the financial institutions' profitability, the possibility of differentiating good applicants from bad ones correctly is extremely important and urgent [3]. Even we can conclude that it is significant to make the accurate credit granting decision because any little improvement such as even a percent fraction in accuracy could be converted into a large future saving for the financial institutions [4].

In general, credit classification approach is used to classify applicants (including individuals and companies) into either good (with credit accepted decisions) or bad (with credit rejected decisions). It is based on the applicants' information such as individual's monthly income, bank balance, vocation, family status, educational background and company's balance sheets, financial ratios, and capital mobility. In detail, good applicants are creditworthy and more capable of repaying loan, bad applicants are not creditworthy, and their capability of loan repayment is low. Consequently, credit analysts have to undertake the responsibility to gather and analyze the relevant information about the loan applicants [5]. As we know, proper credit classification model can reduce credit analysis costs, provide quicker decisions, and reduce potential risk [6]. However, credit classification is a tough task because it is difficult to separate the credit data correctly by using the ordinary approach [7]. The properties of the credit assessment always cause heterogeneity and asynchrony of the information transmitted to the applicants and analysts [8]. Therefore, credit classification always results in high misclassification rate beyond the extent that people normally consider acceptable.

Credit classification techniques are usually estimated through three properties, namely, accuracy, interpretability, and computational efficiency [9]. Accuracy is the essential requirement which represents that the maximum possible number of correct decisions can be generated. And a minor improvement of accuracy means a significant saving for a financial institution. The interpretability is quite important to not only decision makers but also credit applicants, since it represents the ability to generate an understandable evaluation mechanism to the applicants, which includes the choice of the most essential input attributes of the analysis model in the meantime. The computational efficiency represents the speed of classification. It is helpful for the assessors to make the decision whether credit should be granted or not as quickly as possible according to the classification result. Therefore, the credit classification model which owns the above-mentioned properties can be considered as an appropriate tool in the business and finance fields, especially under the conditions with high uncertainty.

Till now, based on particular computation patterns, several models which include artificial neural networks (ANNs) [10–12], decision tree [13], expert systems [14], and genetic algorithms [15, 16] have been proposed to classify bank credit applicants. Among them, the adoption of ANNs in bankruptcy prediction has been studied since the 1990s [17–19]. In 1991, it was the first time for a literature using the neural network to set up the credit classification model and the relative analysis [10, 20, 21]. During the similar period, ANNs have been applied to credit risk assessment and consumer credit scoring research [10, 20, 21]. According to the previous literatures, the general conclusion has been that ANNs outperformed conventional statistical algorithms and inductive learning methods [22]. However, as credit classification methods, the classical ANNs still suffer from the following disadvantages: firstly, they are easy to be trapped into local minimum; thus, sometimes they will cause extraordinarily incorrect experimental results to the financial decision makers and lead to extremely unsatisfactory choices, which will bring a lot of risks for the financial institutions to make the appropriate credit decisions and better investment decisions [23]. Secondly, they are often referred to as black boxes because it is difficult to interpret how the results are concluded [6]. In other words, ANNs are lack of interpretability. Last but not least, most ANNs studies thus far have adopted only limited datasets because when dealing with the high-dimensional credit classification problems, the structures of ANNs are quite large which makes ANNs become time-consuming and thus influence the timeliness of financial decisions [6].

Besides, Lim and Sohn have argued that a single classification algorithm is ineffective because it is not capable of distinguishing the slight differences of various customers [24]. Therefore, in their opinions it is necessary to introduce hybrid classification algorithms to credit classification so as to make the right judgement. However, in terms of average prediction accuracy, it is worth mentioning that some scholars have pointed out that multiple neural networks classifiers are not always superior to a single best neural networks classifier in many cases [25]. This offers us the inspiration to apply a single neural network model to make credit classification.

In order to overcome the above shortcomings of ANNs, we propose a pruning neural network (PNN) which is inspired by the synaptic nonlinearity of biological neural models. It has three layers. Firstly, the axons of the other neurons transform the signals to the synaptic layer; then the interaction of the synaptic signals transfers to every branch of the dendrites; afterwards, the outputs of the dendritic layer are collected and sent to the soma body. Since PNN is a feed-forward neural model, we adopt the error back-propagation algorithm to train it. During the training process, PNN owns the neuronal pruning function to eliminate the unnecessary synapses and superfluous dendrites; thereafter a tidy dendritic morphology will be formed without sacrificing the classification accuracy. Furthermore, we replace the final dendritic structure by logic circuits which are in the form of comparators and logic NOT, AND, and OR gates. Thus, the results of applying PNN on credit classification can be easily implemented in hardware. And fast computational speed will make PNN become a suitable tool for the financial institutions. Experiments are conducted based on Australian and Japanese credit datasets and the results verify that PNN can classify the credit applicants effectively and efficiently in terms of accuracy rate, sensitivity, specificity, and the area under the operating characteristic curve (AUC).

The remaining of the paper is constructed as follows.  Section 2 presents a review of the relative algorithms in credit classification.  Section 3 introduces the proposed neural model PNN in detail. The learning algorithm of PNN is described in Section 4.  Section 5 presents the experimental results of PNN in comparison with other algorithms through using UCI machine learning datasets, namely, Australian and Japanese credit datasets.  Section 6 is dedicated to the discussion.  Section 7 concludes this paper.

## 2. Related Works

**Figure 1:**
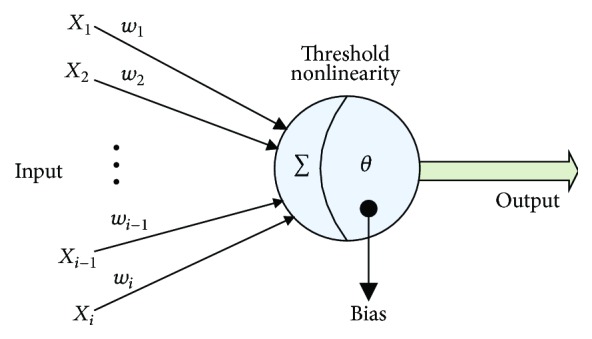
McCulloch-Pitts neuron model.

In the modern studies of neural networks, the McCulloch-Pitts neuron model has been extensively applied [26]. Concretely, the synapses and dendrites are independent of each other and there is no effect on them from one to the other. In its basic unit, each input vector is multiplied by a weight value, and then the result passes through a threshold gate with nonlinearity (see Figure 1). The prevailing view concluded from the previous biological neural networks' literatures has revealed that the brain has great computational capability because of the complicated connectivity of neural networks which implies that McCulloch-Pitts' model is oversimplified to deal with complicated computation [27]. Various modes of synaptic and dendritic plasticity and nonlinearity mechanisms endue the synapses and dendrites the ability to play a significant role in the computation [28]. Individual neuron could act more powerfully whenever the synaptic nonlinearities in a dendrite tree are considered [29–32]. Furthermore, recent research has identified that the already-known neurons all hold a unique shape of dendrite tree [33]. And a small morphological difference would result in a large functional variation. Mode-specific dendritic morphology has its important functional implication in determining what signals a neuron would receive and how these signals would integrate [33].

However, dendritic computation mechanism can provide concrete explanation on targeting the synaptic inputs at the appropriate locations [34]. To be more exact, in the early stage, redundant synapses and dendrites are found in the neural system, while the unnecessary ones will soon be filtered out and the rest will be strengthened and fixed, then a ripened neural network function will be formed [35]. These phenomena offer us the train of thought to propose our model.

According to the measurements made by adopting histological theories, Koch et al. have revealed that the interactions between excitatory synapses and inhibitory synapses have strong nonlinearity, and shunting inhibitory inputs can specifically occlude an excitatory input if they locate on the same path to the soma directly [29]. They have posited that the interactions among synapses and the response at the connection point of a branch could be thought of as logic operations [36]. Nevertheless, their model cannot distinguish whether the excitatory or inhibitory synapse is kept, where it is located, and which branch of dendrite needs to be strengthened [37]. Hence, Koch et al. have pointed out that we need a learning algorithm which is based on the plasticity in dendrites to answer the above questions [38]. It is worth mentioning that, in biological pyramids neurons, manifold plasticity mechanisms have been identified [39–41]. It benefits us to understand the role of plasticity in ANNs. In our previous research [42–45], the well-evolved neurons can be approximately substituted by a logic circuit which is simply composed of the so-called comparators and logic NOT (negation), AND (conjunction), and OR (disjunction) gates according to Boolean algebra. Meanwhile, the locations and types of synapses on the dendrite branches will be formulated by learning [46]. And extra and useless synaptic and dendritic connections would be removed so as to enhance the efficiency of the neurological system [47]. These perspectives and research findings are helpful for us to yield a more realistic model which adopts the single neuron computation to solve the linearly nonseparable problems and improve the neuronal pruning mechanism and then apply it to solve some practical problems such as classifying the credit applicants.

## 3. Proposed Model

**Figure 2:**
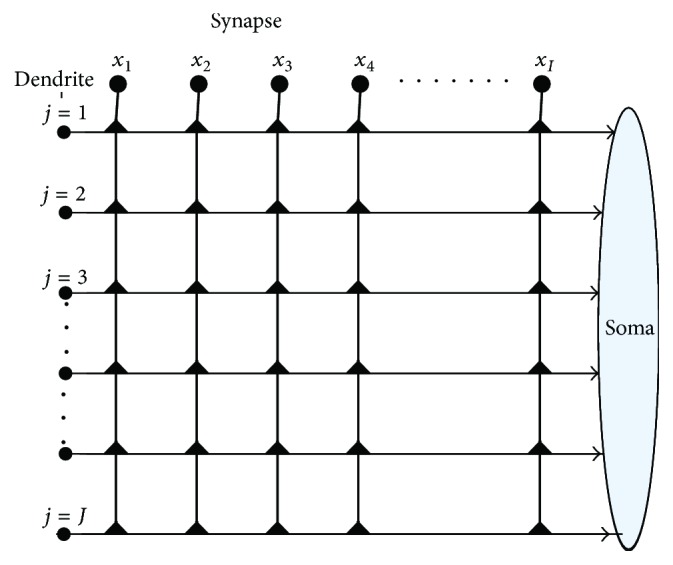
The architecture of PNN.

Inspired by biological neurons, we build up a novel single neuronal structure with dendrites, namely, PNN. PNN has three layers: a synaptic layer, a dendritic layer, and a soma layer, which are shown in Figure 2. The inputs (*x*_1_ to *x*_*I*_) which come from the axons of the prior neurons will enter the synaptic layer. Then, the interactions of the synaptic signals occur on each branch of dendrites. After that, the interactions will be collected and sent to the soma body. The mathematical expressions of PNN are depicted as follows.

### 3.1. Synaptic Layer

The synaptic layer is the region where nerve impulses are transmitted and received among neurons, encompassing the axon terminal of a neuron where neurotransmitters are released in response to an impulse. A synaptic connection to the dendrites of a neuron is implemented by its receptors which have a certain pattern of the specific ion. When the receptors receive an ion, the potential of the receptors converts and determines whether the connection synapse is excitatory or inhibitory [55]. The direction of the flow in the synaptic layer is feed-forward, which always begins from a presynaptic neuron to a postsynaptic neuron. And the equation of the *j*th (*j* = 1,2, 3,…, *J*) synaptic layer receiving the *i*th (*i* = 1,2, 3,…, *I*) input is expressed as follows:

(1) $$\begin{array}{c} Y_{ij}=\frac{1}{1+e^{-k\left(w_{ij}X_{i}-q_{ij}\right)}}, \end{array}$$

where *k* denotes a positive constant, *X*_*i*_ is the input of the synapse, and *w*_*ij*_ and *q*_*ij*_ are synaptic parameters that need to be trained. We use *θ*_*ij*_ to represent the threshold of a synaptic layer, which can be calculated in the following:

(2) $$\begin{array}{c} \theta_{ij}=\frac{q_{ij}}{w_{ij}}. \end{array}$$

**Figure 3:**
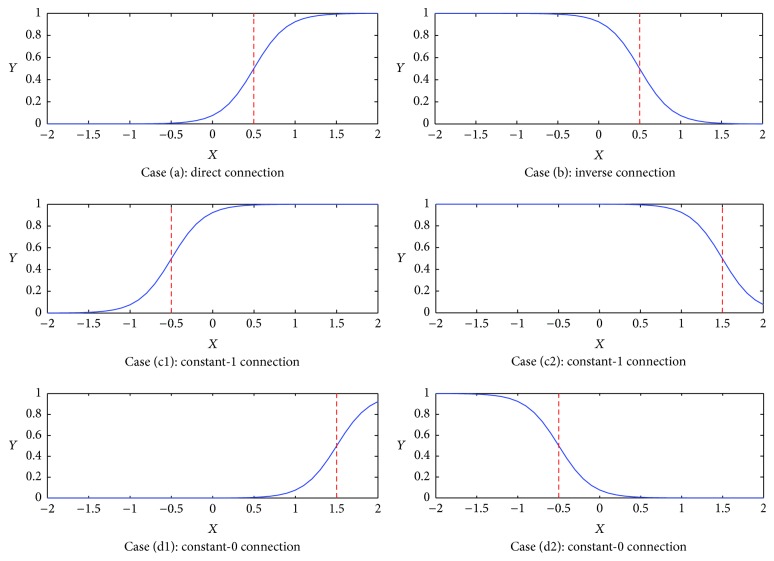
Six connection cases of the synaptic layer.

Depending on different values of *w*_*ij*_ and *q*_*ij*_, there will appear four kinds of connection states: a direct connection, a reverse connection, a constant-1 connection, and a constant-0 connection as shown in Figure 3.

#### 3.1.1. Direct Connection

Case (a): 0 < *q*_*ij*_ < *w*_*ij*_: for example, *q*_*ij*_ = 0.5 and *w*_*ij*_ = 1.0. As shown in Figure 3, Case (a), it corresponds to a direct connection. Once *x*_*i*_ > *θ*_*ij*_, the output *Y*_*ij*_ converges towards 1 which shows that when the input owns higher potential in comparison with the threshold *θ*_*ij*_, the synapse turns to be excitatory which will depolarize the soma body. And when *x*_*i*_ ≤ *θ*_*ij*_, the corresponding output will tend to 0 which represents that once the input possesses low potential, the synapse will change into inhibitory which will hyperpolarize the soma body transiently.

#### 3.1.2. Inverse Connection

Case (b): *w*_*ij*_ < *q*_*ij*_ < 0: for example, *q*_*ij*_ = −0.5 and *w*_*ij*_ = −1.0. As shown in Figure 3, Case (b), it leads to an inverse connection. Once *x*_*i*_ > *θ*_*ij*_, the output *Y*_*ij*_ approximates to 0 which shows that when the input possesses low potential compared with its thresholds, the synapse turns to be inhibitory. And it will hyperpolarize the soma layer transiently. And, on the contrary, when *x*_*i*_ ≤ *θ*_*ij*_ happens, the output *Y*_*ij*_ approximates to 1 which represents that when the input is of high potential, the synapse will become excitatory, and it will depolarize the soma layer.

#### 3.1.3. Constant-1 Connection

There are two cases in the constant-1 connection: Case (c1): *q*_*ij*_ < 0 < *w*_*ij*_: for example, *q*_*ij*_ = −0.5 and *w*_*ij*_ = 1.0. Case (c2): *q*_*ij*_ < *w*_*ij*_ < 0: for example, *q*_*ij*_ = −1.5 and *w*_*ij*_ = −1.0. Case (c1) and Case (c2) shown in Figure 3 represent the constant-1 connection. In these cases, no matter whether the input signal *x*_*i*_ exceeds the threshold *θ*_*ij*_, the corresponding output tends to 1 all the time. In other words, the signals from the synaptic layer have little impact on the dendritic layer. When the excitatory input signals transport, depolarization will occur in the next soma layer.

#### 3.1.4. Constant-0 Connection

The constant-0 connection also contains two cases: Case (d1): *w*_*ij*_ < 0 < *q*_*ij*_: for example, *q*_*ij*_ = 0.5 and *w*_*ij*_ = −1.0. Case (d2): 0 < *w*_*ij*_ < *q*_*ij*_: for example, *q*_*ij*_ = 1.5 and *w*_*ij*_ = 1.0. Case (d1) and Case (d2) illustrated in Figure 3 denote the constant-0 connection. Regardless of the numeric value of the input, the outputs always approximate to 0. The signals from the synaptic layer always degenerate the output signals into an inhibitory one.

**Figure 4:**
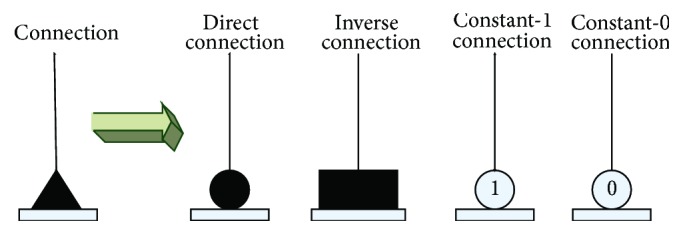
Four connection states of the synaptic layer.

The values of *w*_*ij*_ and *q*_*ij*_ are initialized randomly between −1.5 and 1.5. It means that the synapses are connected to each dendritic branch with randomly chosen connection cases. After being trained by learning algorithms, the values of *w*_*ij*_ and *q*_*ij*_ are changed, and the corresponding connection case of synapses will be changed at the same time.  Figure 4 shows these four connection cases of synapses in our model's structure: a direct connection (•), an inverse connection (■), a constant-1 connection (*①*), and a constant-0 connection (*⓪*).

### 3.2. Dendrite Layer

A dendrite layer stands for the typical nonlinear interaction of synaptic signals on each branch of dendrites. Since the multiplication operation plays an important role in the process of transferring and disposing neural information, the nonlinearity calculation among the synapses on a dendrite can be implemented by a typical multiplication instead of summation. Thus, the interaction among synapses on a dendritic branch corresponds to a logic AND operation. The corresponding equation of the dendrite layer is defined as follows:

(3) $$\begin{array}{c} Z_{j}=\overset{I}{\underset{i=1}{\prod }}Y_{ij}. \end{array}$$

### 3.3. Soma Layer

A soma layer accumulates the summation of the dendritic signals from each dendritic layer. Its function is thought to be the same as a logic OR operation approximately. This logic OR operation implies that the soma body will generate the value 1 when at least one of the variables is equal to 1. Its equation is shown as follows:

(4) $$\begin{array}{c} O=\overset{J}{\underset{j=1}{\sum }}Z_{j}. \end{array}$$

### 3.4. Neuronal Pruning Function

Pruning technique means the removal of the superfluous nodes and weights through learning and training the neural network [56]. In our neural model, pruning function can be achieved by eliminating unnecessary synapses and dendrites. And a simplified and unique neural structure will be formed for each specific problem. Neuronal pruning function of our model contains two parts: synaptic pruning and dendritic pruning.

*Synaptic Pruning*. When the input transmits to the synaptic layer which is in the constant-1 connection case, the synaptic output is always 1, because the result of any arbitrary value multiplying 1 equals itself in the dendrite layer. It is obvious that the synaptic input in constant-1 connection has little impact on the output of the dendrite layer. Therefore, this kind of synaptic input could be absolutely neglected.

*Dendritic Pruning*. If the input transmits to the synaptic layer which is in the constant-0 connection case, the output is always 0. Consequently, the output of the corresponding dendrite layer also becomes 0 because of the multiplication operation. It means that this entire dendrite layer should be omitted because it has little influence on the soma layer.

**Figure 5:**
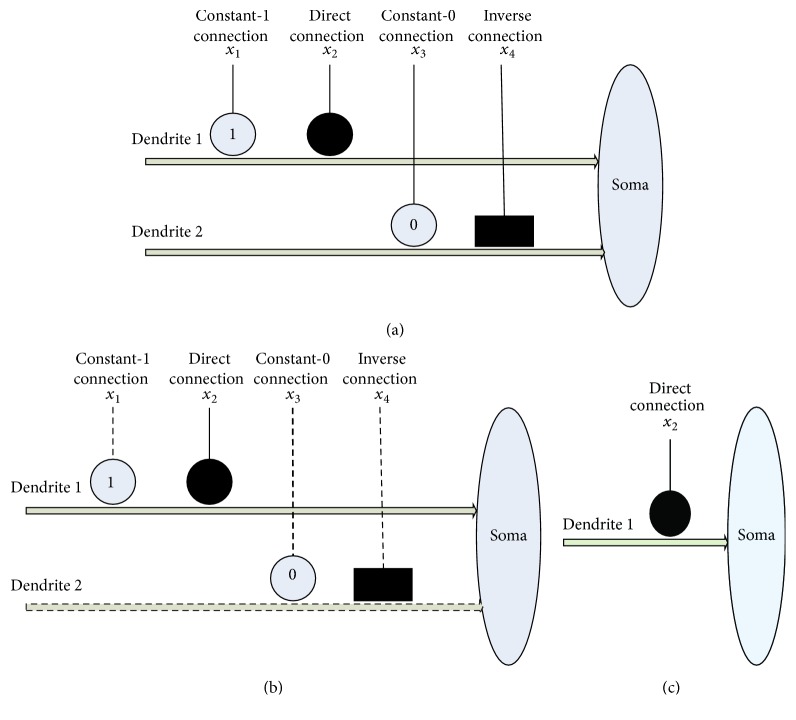
An example of a synaptic and dendritic pruning procedure.

An example of a synaptic and dendritic pruning procedure is illustrated in Figure 5. The original structure is composed of four synaptic layers and two dendrite layers as shown in Figure 5(a). Since the connection case of input *x*_1_ is *①* in Dendrite-1 layer, this synaptic layer can be deleted. The connection case of input *x*_3_ is *⓪* in the Dendrite-2 layer; the whole Dendrite-2 can be completely omitted because of the dendritic pruning function. The unnecessary synaptic layers and dendrite layers which could be removed are shown in dotted lines as shown in Figure 5(b). The final simplified dendritic morphology is shown in Figure 5(c), in which only a synaptic layer and a dendritic layer are retained.

## 4. Learning Algorithm

As all the equations of PNN are differential, the error back-propagation algorithm (BP) is valid to be utilized as the learning algorithm. The BP algorithm adjusts the values of *w*_*ij*_ and *q*_*ij*_ to cut down the differences between the actual output *O* and desired output *T*. The Least Squared Error (LSE) between the actual output and desired output is defined in (1):

(5) $$\begin{array}{c} E=\frac{1}{2}{\left(\;T-O\;\right)}^{2}. \end{array}$$

In PNN, the error minimization is realized by modifying the connection parameters in the negative gradient direction during the learning process. Hence, the differential changes of these connection parameters should be collected as shown in the following equations:

(6) Δwij=−η∂E∂wij,Δqij=−η∂E∂qij,

where *η* denotes the learning rate and it is always set to be a positive constant. However, a low learning rate makes the convergence speed very slow, whereas a high learning rate makes the error become very difficult to converge to a certain connection pattern. The updating rules for connection parameters *w*_*ij*_ and *q*_*ij*_ are as follows:

(7) wijt+1=wijt+Δwijt,qijt+1=qijt+Δqijt,

where *t* represents the current learning epoch. Moreover, the partial differentials of *E* with respect to *w*_*ij*_ and *q*_*ij*_ are computed in the following:

(8) ∂E∂wij=∂E∂O·∂O∂Zj·∂Zj∂Yij·∂Yij∂wij,∂E∂qij=∂E∂O·∂O∂Zj·∂Zj∂Yij·∂Yij∂qij.

The following shows the components of the above-mentioned partial differential:

(9) ∂E∂O=O−T,∂O∂Zj=1,∂Zj∂Yij=∏L=1,L≠iIYLj,∂Yij∂wij=kxie−kXiwij−qij1+e−kXiwij−qij2,∂Yij∂qij=−ke−kXiwij−qij1+e−kXiwij−qij2.

## 5. Simulation

### 5.1. Credit Dataset Description

In this experiment, we have adopted two benchmark datasets, namely, the Australian and Japanese credit datasets (all from the UCI repository), to test different classification models. With a good mixture of different attributes which includes not only continuous but also nominal attributes with both small and large numbers of values, these two real world datasets are very meaningful to financial decision makers and managers. The details of the attributes can be found from the UCI repository [57].

Australian credit dataset is used to classify credit card applications and it contains 690 examples that record the applicants' data. This dataset contains 307 examples of creditworthy applicants (“good” and “accepted”) and 383 examples which are not creditworthy (“bad” and “rejected”). Each instance consists of 8 categorical and 6 numerical input attributes. In order to protect the secrecy of the credit applicants, the applicants' names and values of the attributes have been converted to meaningless symbols.

Japanese credit dataset also contains 690 instances, which are classified into two groups. Among them, 307 are labeled as class “+” and the rest 383 are labeled as “−.” And each sample is characterized by 6 numerical and 9 categorical features. Similar as Australian credit dataset, the applicants' names and the attribute values have been converted to meaningless symbols to protect the data confidentiality.

### 5.2. Data Preprocessing

Data preprocessing is the first and crucial step to make data analysis. The classification task would be misleading and redundant if the data are not understood and considered completely in advance. Firstly, it sometimes shows a few missing values in the dataset, and the majority of learning algorithms are lack of the ability to handle the datasets with missing values. It needs us to utilize some methods to replace them [58]. In our experiments, we replace the numerical attribute with the average values and categorical ones with the mode of attributes, respectively.

Secondly, some learning algorithms such as ANNs require that each data sample is expressed as a real number vector. Thus, we need to transform the categorical attributes into numerical ones before we input them into the classifier. The attribute information of Australian credit dataset has been changed for the convenience of statistics. For example, the fourth attribute of this dataset has 3 labels, namely, “p,” “g,” and “gg.” And these labels have been changed to 1, 2, and 3 in our experiments. According to this method, all the categorical attributes of Australian credit dataset and Japanese credit dataset have been changed, which are presented in Tables 1 and 2, separately.

Last but not least, for the sake of preventing the large numerical attributes from dominating those with small numerical values, all the numerical values should be normalized. In general, all the attributes are normalized to a range of [0,1] with a min-max normalization rule. And the min-max normalization procedure uses a linear transformation to change the original input range into a new specified range, which can be shown in the following equation:

(10) $$\begin{array}{c} x_{normalized}=\frac{x-x_{min}}{x_{max}-x_{min}}. \end{array}$$

### 5.3. Performance Measures

In our experiments, overall accuracy rate, true positive rate, true negative rate, and AUC which is the area under the receiver operating characteristic curve (ROC) are utilized to construct the performance evaluation system. Firstly, the classification accuracy rate is regarded as one of the most popular classification performance metrics. It is measured by using the following equation:

(11) $$\begin{array}{c} Accuracy\;\;rate=\frac{TP+TN}{TP+TN+FP+FN}\left(\;\%\;\right), \end{array}$$

where TP, TN, FP, and FN represent true positive, true negative, false positive, and false negative, respectively. True positive (TP) indicates the number of the instances which are predicted as creditworthy and their corresponding teacher target labels are creditworthy too. True negative (TN) represents the number of the instances whose prediction label and teacher target label are uncreditworthy at the same time. And false positive (FP) denotes the number of the samples which are detected as uncreditworthy, while the teacher target label is creditworthy. On the contrary, false negative (FN) stands for the number of the samples which are detected as creditworthy, but their teacher target labels are not. The results of a classifier containing TP, TN, FP, and FN can be measured by a 2-dimensional contingency matrix, which is demonstrated in Table 3.

Sensitivity and specificity are also important performance metrics in classification problems. Sensitivity measures the percentage that actual positives are correctly identified. It implies how successfully a classifier can identify the normal records which means that the applicants are creditworthy in the case of credit classification. Therefore, financial institutions can reduce their possible financial losses by adopting the classifier with higher sensitivity. Specificity measures the number of the observed bad applicants occupying a certain proportion of the total number of the observed bad applicants and those classified as bad. Thus, it represents how successfully a classifier can distinguish the abnormal records, so it means the proportion of true negative. Higher specificity can help the financial institutions to reduce the possibility of accepting the applicants with bad credit. And the expressions of sensitivity and specificity are shown as follows:

(12) Sensitivity=TPTP+FN%,Specificity=TNTN+FP%.

In this study, AUC is also designed as significant metric to evaluate the model. And it can be calculated from the graph in which the sensitivity is plotted on the *y* axis and specificity is plotted on *x* axis, respectively. AUC reveals the difference between the classification groups predicted by a classifier. In other words, a score of 100% indicates that two classes can be perfectly discriminated by the classifier, while a score of 50% illustrates that the classifier owns insignificant discriminatory quality. The value of AUC can be demonstrated as follows:

(13) $$\begin{array}{c} AUC\;\left(\;\%\;\right)=\frac{1}{2}\left(\;\frac{TP}{TP+FN}+\frac{TN}{TN+FP}\;\right)\times 100\;\left(\;\%\;\right). \end{array}$$

Besides, in order to compare the convergence speed of different classification algorithms, the mean squared error (MSE) of PNN and MLP at each iteration is calculated by the following equation:

(14) $$\begin{array}{c} MSE=\frac{1}{R}\overset{R}{\underset{a=1}{\sum }}\left[\;\frac{1}{S}\overset{S}{\underset{b=1}{\sum }}{\left(\;E_{ab}-O_{ab}\;\right)}^{2}\;\right], \end{array}$$

where *E*_*ab*_ and *O*_*ab*_ represent the predicted output and the actual output separately. *S* is the number of instances applied for training. *R* denotes the running times of the experiments which is set to be 30 to classify both Australian and Japanese credit datasets in our experiments.

### 5.4. Optimal Parameters Setting

Three user-defined parameters are considered to be sensitive to the classification performance of PNN, namely, *k*, *η*, and *M*. *k* represents a constant of the sigmoid function in the synaptic layer, *η* denotes the learning rate, and *M* means the branch number of the dendritic layer. It is necessary to determine an optimal set of parameters to obtain high accuracy rate and fast convergence speed. Thus, we employ the Taguchi method to produce the orthogonal arrays [59], which can reduce the number of trails to control the cost of time, manpower, and materials effectively. Each parameter is defined to own four levels in PNN. We provide *L*_16_(4^3^) orthogonal arrays for both benchmark datasets, which are illustrated in Tables 4 and 5. The corresponding accuracies of PNN with each parameter set are also shown in these tables. We can find that, for Australian credit dataset, the parameter set of the 3rd row (*k* = 2, *η* = 0.08, *M* = 30) has better performance than the other sets. And the highest testing accuracy of Japanese credit dataset occurs on the 8th row (*k* = 2.5, *η* = 0.07, *M* = 30). These parameter combinations are reasonable to obtain acceptable performance; to some extent they reveal the effects of the parameters on the performance of PNN. These parameter sets are reasonable to obtain acceptable performance for two benchmark datasets, and we use these parameter sets as the optimal ones to make further comparison with the other classifiers in our experiments.

In our experiments, PNN is compared with the classical multilayer perceptron (MLP) to solve both benchmark problems. PNN and MLP have different neuronal structures, but they utilize the same learning algorithm. For a relatively fair comparison, the number of weights and thresholds of both models should be approximately equal, which can be calculated as follows:

(15) $$\begin{array}{c} N_{MLP}=I\times L+2L+1, \end{array}$$

where *N*_MLP_ denotes the amount of the relevant weights and thresholds which need to be adjusted in the structure of MLP. *I* represents the number of neurons in the input layer. And *L* means the neuron numbers in the hidden layers.

(16) $$\begin{array}{c} N_{PNN}=2I\times J, \end{array}$$

where *N*_PNN_ refers to the number of the relevant weights and thresholds which need to be adjusted in the structure of PNN. *I* represents the number of synapses on each branch of dendrites. And *J* means the numbers of the dendrite branches. The numbers of the adjusted parameters of both benchmark datasets in our simulation are summarized in Table 6.

### 5.5. Performance Comparison

To evaluate the performances of different classification methods, each dataset is randomly separated into two subsets, one is for training and the other is for testing. The training subset is used to train the classification model, and the testing one is adopted to verify the validity of the model. And the percentages of the training and the testing subset are set to be 50% and 50% [60], respectively. All the experiments of the two benchmark datasets run 30 times, the average (mean) and standard deviation (Std) of the results are provided in the form of Mean ± Std.

In the classification investigation field, cross-validation is widely applied to test the model's robustness, especially under the uncertainty with unknown class labels [61]. In contrast with the single-fold validation method, the multifold cross-validation (CV) such as *K*-fold CV has the advantage to minimize the bias caused by random sampling, whereas it has the disadvantages of excessive computation time and cost requirement [62]. In our experiments, 5-fold CV and 10-fold CV methods are applied to compare PNN with the other classifiers.

In addition, a nonparameter statistical test, namely, Wilcoxon rank-sum test was adopted to detect the significant difference between PNN and MLP in our experiments. The null hypothesis means that there is no difference between two models, and the required significance level is set to be 0.05. If the *p* value is less than 0.05, there is a strong evidence to reject the null hypothesis. And if it is larger than 0.05, the null hypothesis cannot be rejected. N/A represents “Not Applicable” which indicates that the relevant algorithm does not need to be compared with itself.

#### 5.5.1. Australian Credit Dataset

In this section, PNN is firstly compared with MLP to solve Australian credit problem. The learning rate is set to be 0.08 for both models. As shown in Table 7, the proposed PNN acquires an average testing accuracy of 85.64%, which is higher than the 84.23% accuracy rate obtained by MLP. The *p* value of Wilcoxon rank-sum test is 0.0038, which is smaller than the required significance level (0.05). It implies that there is a significant difference between PNN and MLP to solve Australian credit problem. Moreover, PNN also performs better than MLP in the aspects of sensitivity and specificity. Convergence speed is also a performance metric which affects the efficiency of a model. The convergence curves of PNN and MLP for Australian credit dataset are compared in Figure 7. It can be observed that, at the beginning, the convergence speed of MLP is higher than that of PNN, while PNN converges more quickly since the 50th iteration of the training process.

**Figure 6:**
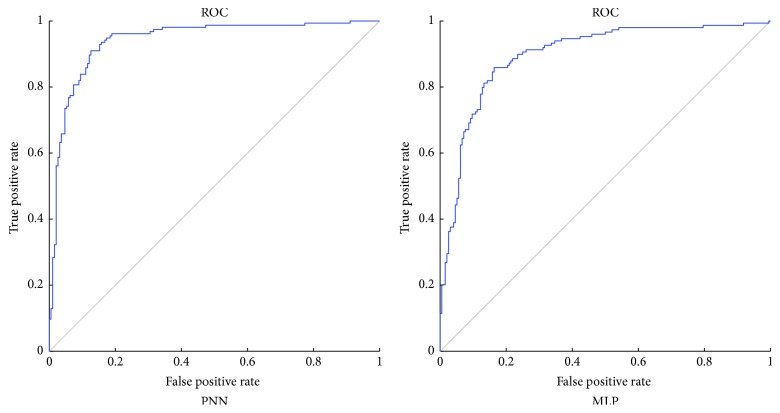
The ROC of PNN and MLP for Australian credit dataset.

**Figure 7:**
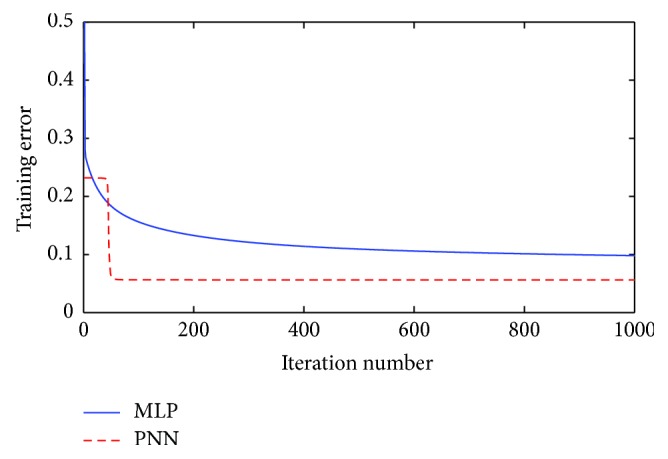
Comparison of convergence speed between PNN and MLP of Australian credit dataset.

Based on the sensitivity and specificity values of PNN and MLP in Table 7, we can conclude that a higher sensitivity value indicates that PNN is more powerful to identify the applicants who are creditworthy. And a higher specificity value represents that PNN has a smaller probability to misjudge a creditworthy applicant when solving Australian credit problem.  Figure 6 shows the ROC curves of PNN and MLP. By calculating the area under the curves, the AUC value of PNN (0.9411) is found to be larger than that of MLP (0.8976). Besides MLP, we compare PNN with some other classifiers, such as support vector machine (SVM), *K*th nearest neighbor (KNN), and Bayesian network. The corresponding results have been illustrated in Table 8. We can find that all the three cases of PNN, namely, 50%-50%, 5 × CV, and 10 × CV, have performed higher classification accuracy rates than the other classifiers. It has once again proved that PNN is capable of providing superior performances to solve Australian credit problem.

#### 5.5.2. Japanese Credit Dataset

**Figure 8:**
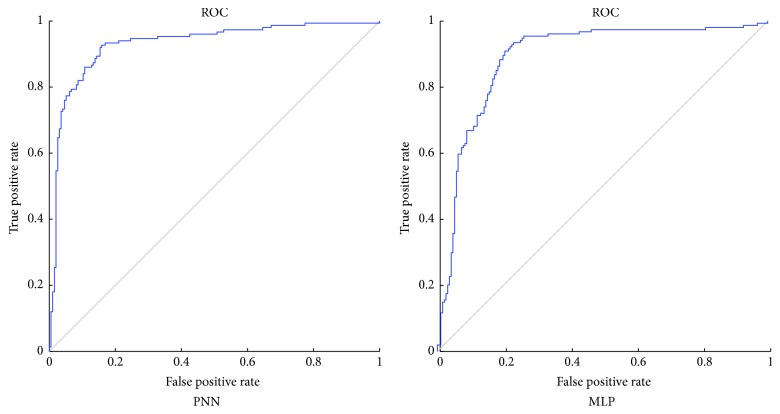
The ROC of PNN and MLP for Japanese credit dataset.

**Figure 9:**
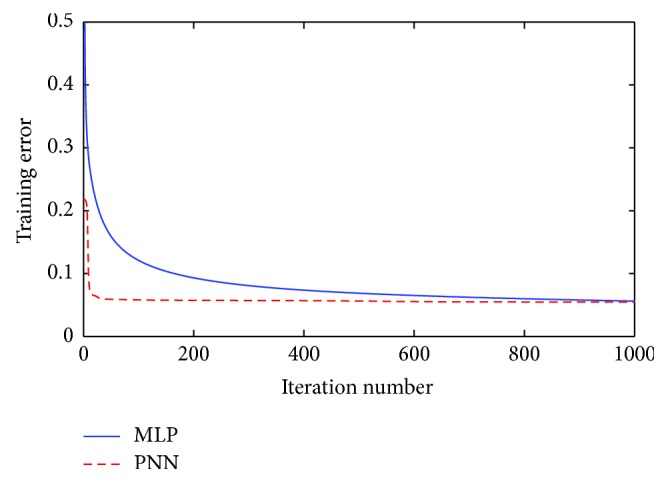
Comparison of convergence speed between PNN and MLP of Japanese credit dataset.

When dealing with the Japanese credit dataset, learning rate of both PNN and MLP is set to be 0.07. As shown in Table 9, the average testing accuracy rate of 30 times experiments of PNN is 85.54%, which is higher than that of MLP. *p* value of Wilcoxon test is 6.4811*e*^−05^, and it is smaller than the required significance level (0.05). Thus, we can conclude that the accuracy of PNN is significantly higher than that of MLP. What is more, PNN obtains higher values of sensitivity and specificity than MLP, which implies that PNN is more powerful to retain creditworthy applicants and remove uncreditworthy applicants, when dealing with Japanese credit problem. It also can be observed from the ROC curves, which are illustrated in Figure 8. By calculating the area under ROC, we can find that the AUC of PNN is 0.9301, which is larger than that of MLP. The convergence curves of PNN and MLP are provided in Figure 9. As it is observed, PNN converges very quickly and nearly achieves the best convergence performance at the 20th iteration. At the end of the training process, PNN presents lower training error than MLP.

In addition, we compare the classification performance of PNN with some other classifiers, and the comparison has been summarized in Table 10. The accuracies of three cases of our method (50%-50%, 5 × CV, and 10 × CV) are 85.54%, 85.23%, and 85.27%, respectively. All of them are obviously higher than the other classifiers. Based on these results, it can be concluded that PNN possesses a relatively high convergence speed and accuracy to solve Japanese credit problem.

### 5.6. Dendrite Morphology Reconstruction

#### 5.6.1. The Ultimate Synaptic and Dendritic Morphology

As mentioned above, PNN utilizes synaptic pruning and dendritic pruning to realize structural plasticity, and the superfluous synapses and useless dendrites can be removed during the process of learning. Hence, a simplified and distinct structural morphology is formed and it can be replaced by a logic circuit. In this section, we verify the effectiveness of the neuronal pruning function and the accuracy of the logical circuit by applying Australian and Japanese credit datasets.

**Figure 10:**
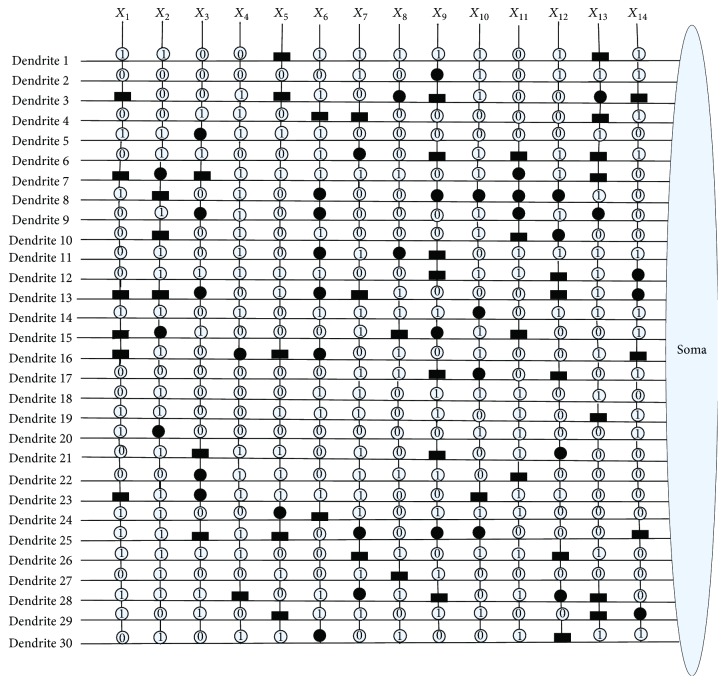
The dendritic morphology of the Australian credit dataset before learning.

**Figure 11:**
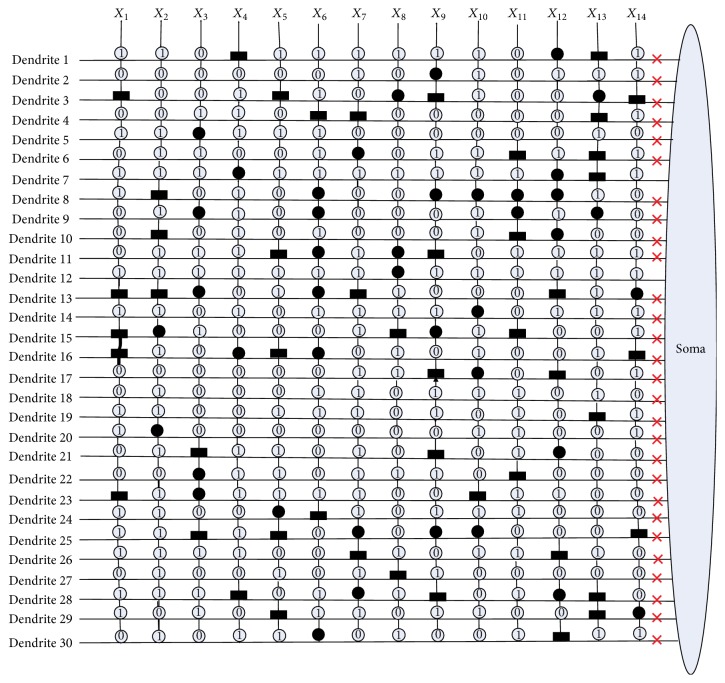
The dendritic morphology of the Australian credit dataset after learning.

**Figure 12:**
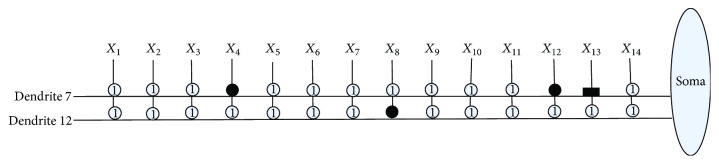
The dendritic morphology of the Australian credit dataset after dendritic pruning.

**Figure 13:**
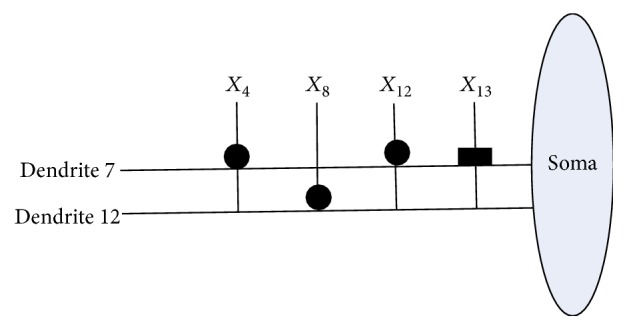
The dendritic morphology of the Australian credit dataset after synaptic pruning.

Figure 10 shows the dendritic structure of Australian credit dataset before learning. As it shows, there are 30 branches of dendrites in the structure, and each branch has 14 synapses which connect to 14 input features. All the connection cases of these synapses are determined by the randomly chosen weights and thresholds.  Figure 11 presents the relative structure after learning. We use the symbol “×” to represent that this dendrite can be removed by dendritic pruning.  Figure 12 shows that all the unnecessary branches of dendrites which own the synapses in constant-0 connection case are detected; only dendrite 7 and dendrite 12 are retained. After removing all the synapses in the constant-1 connection cases, the final synaptic and dendritic morphology is described in Figure 13, and it can be observed that we delete all the unnecessary synapses which connect to *X*_1_, *X*_2_, *X*_3_, *X*_5_, *X*_6_, *X*_7_, *X*_9_, *X*_10_, *X*_11_, and *X*_14_. The final reserved features are only *X*_4_, *X*_8_, *X*_12_, and *X*_13_ for Australian credit dataset.

**Figure 14:**
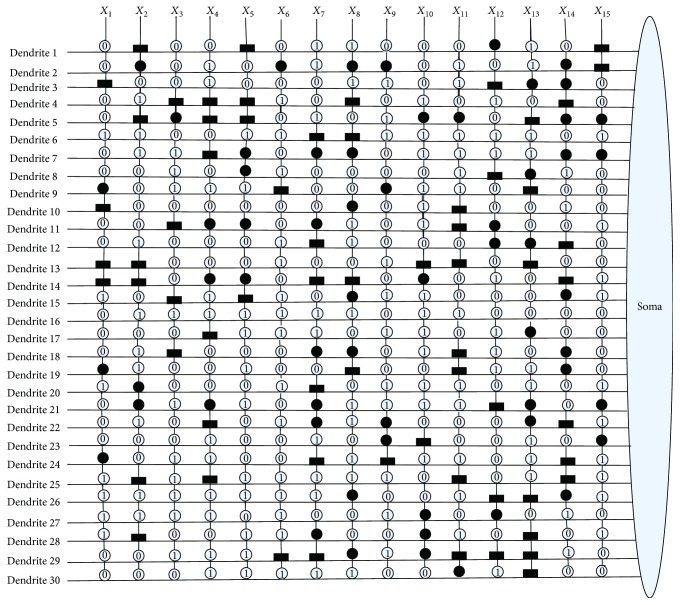
The dendritic morphology of the Japanese credit dataset before learning.

**Figure 15:**
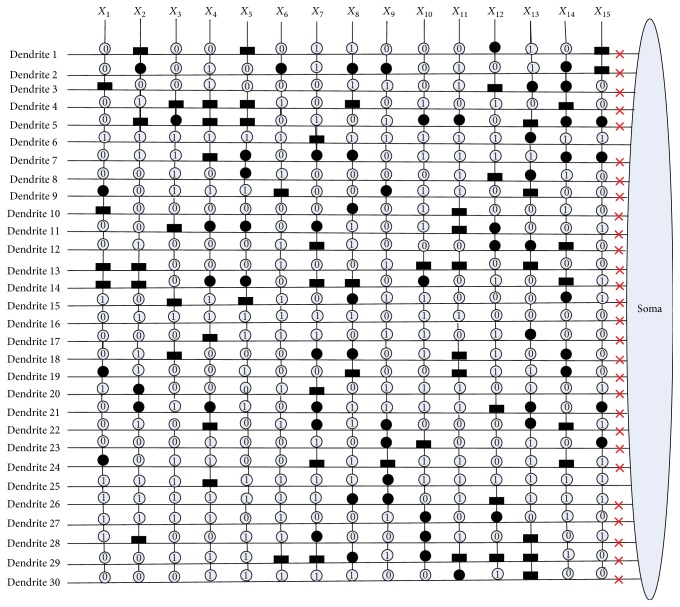
The dendritic morphology of the Japanese credit dataset after learning.

**Figure 16:**
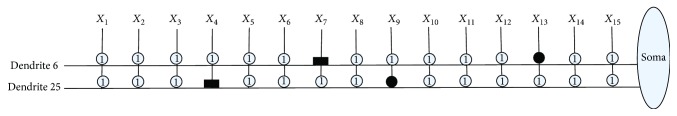
The dendritic morphology of the Japanese credit dataset after dendritic pruning.

**Figure 17:**
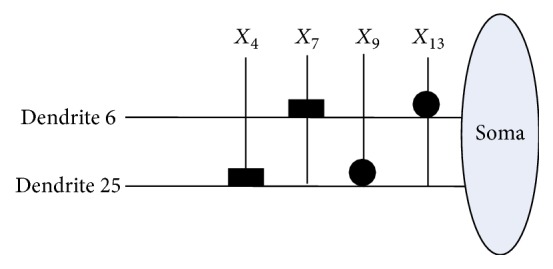
The dendritic morphology of the Japanese credit dataset after synaptic pruning.

Then, the same process of disposing Japanese credit dataset has been illustrated in Figures 14, 15, 16, and 17. It can be observed that the neuronal pruning function has totally abandoned 28 unnecessary branches of dendrites and 11 redundant features. Only features *X*_4_, *X*_7_, *X*_9_, and *X*_13_ are reserved in the final structure of PNN. The model structure comparison between Australian and Japanese credit datasets is summarized in Table 11.

#### 5.6.2. The Simplified Logic Circuit (LC) of the After-Learning Morphology

**Figure 18:**
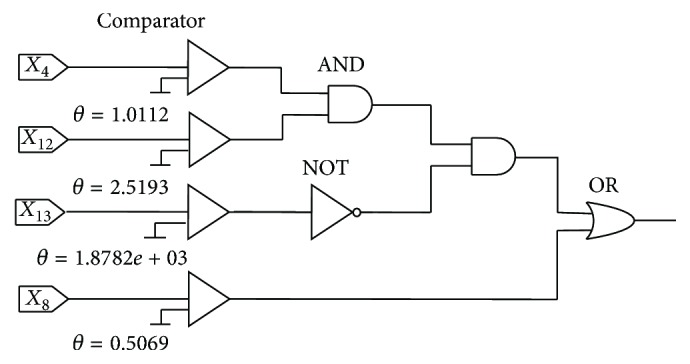
LC of the Australian credit dataset.

**Figure 19:**
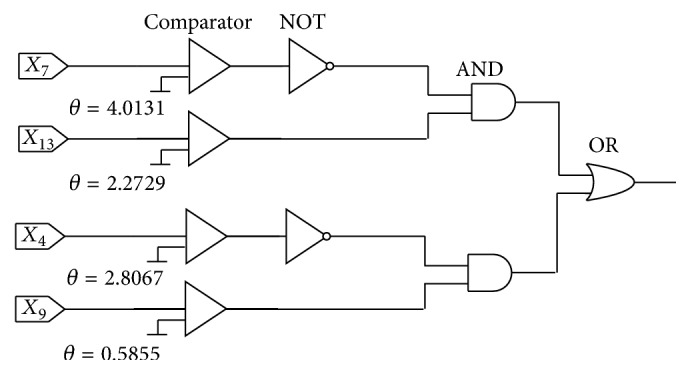
LC of the Japanese credit dataset.

After implementing the neuronal pruning function, we have obtained two simplified model structures for both benchmark problems. Then, these models are replaced by logic circuits (LCs) which consist of an analog-to-digital converter, namely, “comparator,” logical “NOT,” “AND,” and “OR” gates. LCs of both benchmark datasets are presented in Figures 18 and 19, respectively. A comparator is used to compare the practical input with the threshold *θ*. Once the input *x*_*i*_ is less than the threshold *θ*, the “comparator” will output 0. On the contrary, if the input exceeds the threshold *θ*, the output will be 1. By these LCs, we can classify the applicants into accepted and rejected for the Australian credit dataset and Japanese credit dataset. Moreover, we calculate the accuracy of these LCs and provide the results in Table 12. It is obvious that the test accuracy of the Australian credit dataset is 85.80%, and the test accuracy of the Japanese credit dataset is 85.51%. They are nearly equal to the accuracies of PNN before simplification which are 85.64% and 85.54%.

**Figure 20:**
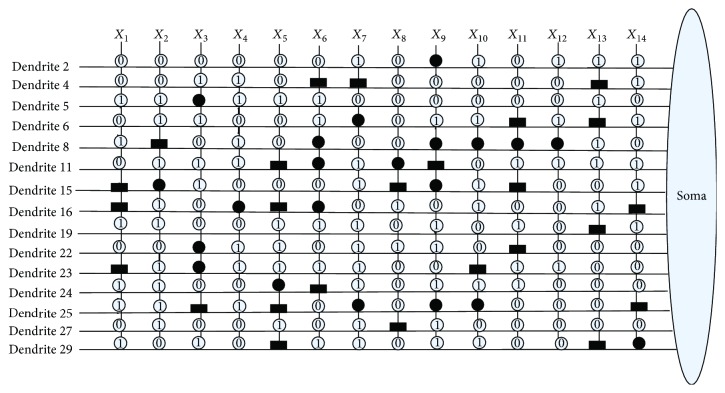
The simplified structure of the Australian credit dataset acquired by CP.

**Figure 21:**
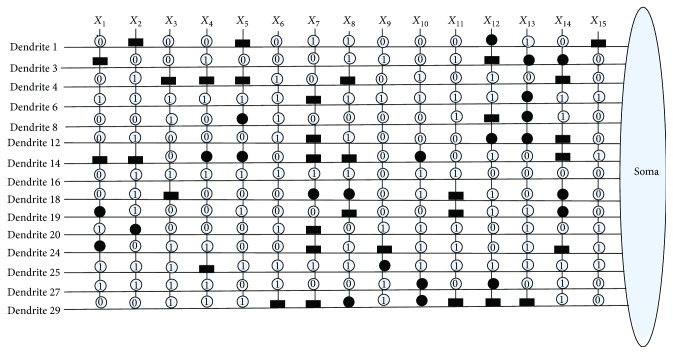
The simplified structure of the Japanese credit dataset acquired by CP.

Moreover, we compare the results of another pruning method (named as “correlation pruning (CP)”) to simplify the PNN structure. Specifically, the method detects the pair of the most highly correlated branches of dendrites in the morphology structure. Each dendritic branch is represented by the vector *B*_*i*_ which consists of its synaptic parameters *w*_*ij*_ and *q*_*ij*_ in the *i*th dendritic branch. Then, one of the dendritic branches in the pair will be deleted randomly. The process repeats until the branch number of PNN satisfies a predetermined number *pn*. In our experiments, the values of *pn* of the Australian and Japanese credit datasets are set to be 15, and both experiments are run 30 times independently. The correlation coefficient *r* is defined as follows:

(17) $$\begin{array}{c} r\left(\;B_{i},B_{j}\;\right)=\frac{\mathrm{cov}\left(B_{i},B_{j}\right)}{\sqrt{\mathrm{var}\left[B_{i}\right]*\mathrm{var}\left[B_{j}\right]}}, \end{array}$$

where cov⁡(*B*_*i*_, *B*_*j*_) represents the covariance of *B*_*i*_ and *B*_*j*_ and var⁡[*B*_*i*_] and var⁡[*B*_*j*_] denote the variance of *B*_*i*_ and *B*_*j*_, respectively. Figures 20 and 21 illustrate the simplified structures of PNN, and CP discards 15 highly correlated branches for both datasets. The average of the test accuracy rate of these simplified PNN structures is also presented in Table 12. It can be observed that the test accuracy rates of the Australian and Japanese credit datasets are 61.21% and 65.56%, respectively. They are smaller than the results of PNN and the simplified LCs. It means that the replacement of LCs is more effective, and it does not sacrifice the classification accuracy rate of PNN. In addition, LCs can be easily implemented in hardware, which can achieve a high computational speed. Based on these excellent characteristics, the obtained LCs are thought to be powerful classifiers for the benchmark datasets.

## 6. Discussion

Based on the above experimental results, the following is clear. Firstly, PNN has higher accuracy rate than MLP on both benchmark datasets. It means PNN can offer much more correct decision support for the financial institutions. Secondly, higher values of sensitivity and specificity imply that PNN is able to retain better applicants and remove worse applicants with a high probability, respectively. Thirdly, PNN has a larger value of AUC which represents that the differences between creditworthy and uncreditworthy groups classified by PNN are more obvious. This point is very important in the credit risk assessment because it is helpful to make the financial institutions and credit applicants accept the classification result more easily. Lastly, PNN provides two LCs for both benchmark datasets. These LCs have satisfactory classification performances and they will extremely speed up the classification to offer correct and quick decision for the decision makers. Although many novel algorithms are constantly emerging to solve the credit classification problems, many approaches have still merely focused on the credit classification models' ability of improving the classification accuracy rate, while PNN provides a brand new perspective to improve the efficiency of ANNs.

Feature selection is one of the most important steps of machine learning in the process of data mining. It focuses on filtering out the redundant and irrelevant features from the original large-scale datasets, which can reduce the running time of a learning algorithm and improve the model's performance consequently as well as reducing the effort of training the model [63]. Many algorithms have been proposed to select effective features from the input attributes such as *t*-test, stepwise, and related matrix [54]. It is worth mentioning that PNN can also implement feature selection during the training process because the pruning function can reduce not only the superfluous branches of dendrites but also the unnecessary synapses. And each synapse connects to the input of a feature. If all these kinds of synapses are eliminated, the features will be extracted. The extraction rates of the two benchmark datasets are shown in Tables 13 and 14, respectively. It can be observed that, for the Australian credit dataset, although the accuracy rate of PNN is not the best one, the feature extraction rate of PNN is obviously higher than the other five methods. As for Japanese dataset, PNN has the highest accuracy rate and extraction rate simultaneously. Therefore, we can conclude that PNN owns the best extraction rate among the six feature selection methods. It is notable that the features selected by PNN are only verified to be effective in our neural model. In our future research, we intend to investigate whether these selected features can remain suitable in other classification algorithms.

It is inevitable that although PNN performs satisfactorily on several aspects, it also has its disadvantages such as its results lack of interpretability especially on analyzing the pruning reasons for the input variables. Interpretability represents whether the classification results can be explained clearly to the applicants [51]. This will be a major drawback and cause a reluctance to use the approach. Even it may go further that when credit application has been refused to a client, the financial institutions should provide definite reasons legally why the application is rejected. Previous literature reviews show that some algorithms perform well in one or two aspects at most but bad in the remaining aspects. In a word, there is nearly no algorithm which can balance accuracy, complexity, and interpretability; PNN is no exception. In order to acquire useful and understandable knowledge, adopting a visual and interactive framework will be an inevitable trend to integrate the users into the black-box process.

## 7. Conclusion

In this paper, a pruning neural network classifier PNN is proposed for credit classification. We can conclude that, in contrast with MLP and other classifiers, PNN performs the best in terms of the average accuracy rate, sensitivity, specificity, and AUC for the two popularly applied benchmark datasets, namely, Australian credit dataset and Japanese credit dataset. Besides, PNN has provided tidy neuronal morphologies and LCs by synaptic and dendritic pruning for both datasets. And the efficiency of LCs has been verified in our experiments. Therefore, PNN will be a very effective and efficient method to solve the classification problems.

In summary, the contributions of this paper are as follows. (1) The PNN model that we have proposed gets further access to the realistic biological neural model in comparison with other ANNs. (2) PNN can simplify its structure during the training process by its synaptic and dendritic pruning mechanisms. (3) PNN settles the credit classification issue efficiently in terms of accuracy and convergence speed. (4) PNN offers another perspective to realize feature selection. (5) LCs of the two classification benchmark problems obtain satisfactory accuracy and higher computation speed. These points imply that the proposed PNN owns great potential to be applied in solving other real world classification problems in the big data era.

## Figures and Tables

**Table 1:** Attributes for evaluating credit risk in the Australian credit dataset.

Attributes	Type	Values (after preprocessing)	Values (before preprocessing)
A1	Categorical	0,1	a, b
A2	Numerical	13.75–80.25	13.75–80.25
A3	Numerical	0–28	0–28
A4	Categorical	1,2, 3	p, g, gg
A5	Categorical	1,2, 3,…, 14	ff, d, i, k, j, aa, m, c, w, e, q, r, cc, x
A6	Categorical	1,2, 3,…, 9	ff, dd, j, bb, v, n, o, h, z
A7	Numerical	0–28.5	0–28.5
A8	Categorical	0,1	t, f
A9	Categorical	0,1	t, f
A10	Numerical	0–67	0–67
A11	Categorical	0,1	t, f
A12	Categorical	1,2, 3	s, g, p
A13	Numerical	0–2000	0–2000
A14	Numerical	0–100,000	0–100,000
Class	Categorical	0,1	−, +

**Table 2:** Attributes for evaluating credit risk in the Japanese credit dataset.

Attributes	Type	Values (after preprocessing)	Values (before preprocessing)
A1	Categorical	0,1	a, b
A2	Numerical	13.75–80.25	13.75–80.25
A3	Numerical	0–28	0–28
A4	Categorical	1,2, 3,4	u, y, l, t
A5	Categorical	1,2, 3	g, p, gg
A6	Categorical	1,2, 3,4,…, 14	c, d, cc, i, j, k, m, r, q, w, x, e, aa, ff
A7	Categorical	1,2, 3,4,…, 9	v, h, bb, j, n, z, dd, ff, o
A8	Numerical	0–28.5	0–28.5
A9	Categorical	1,0	t, f
A10	Categorical	1,0	t, f
A11	Numerical	0–67	0–67
A12	Categorical	1,0	t, f
A13	Categorical	2,3, 1	g, p, s
A14	Numerical	0–2000	0–2000
A15	Numerical	0–100,000	0–100,000
Class	Categorical	0,1	−, +

**Table 3:** Contingency matrix of prediction results.

Hypothesis class	Real class	
	Positive	Negative
Positive	True positive (TP)	False positive (FP)
Negative	False negative (FN)	True negative (TN)

**Table 4:** *L*_16_(4^3^) orthogonal array and factor assignment of the Australian credit dataset.

Expe. number/parameter	*k*	*η*	*M*	Testing accuracy
1	2	0.01	25	85.26 ± 1.13
2	2	0.05	28	85.54 ± 1.27
3	2	0.08	30	85.64 ± 1.74
4	2	0.1	32	84.88 ± 1.59
5	3	0.01	25	84.22 ± 5.68
6	3	0.05	28	85.45 ± 1.56
7	3	0.08	32	84.98 ± 1.73
8	3	0.1	30	85.34 ± 1.66
9	4	0.01	30	82.48 ± 5.55
10	4	0.05	32	82.89 ± 5.26
11	4	0.08	25	83.73 ± 3.35
12	4	0.1	28	83.22 ± 4.27
13	5	0.01	32	74.16 ± 8.85
14	5	0.05	30	76.03 ± 9.87
15	5	0.08	28	73.17 ± 12.33
16	5	0.1	25	71.03 ± 12.68

**Table 5:** *L*_16_(4^3^) orthogonal array and factor assignment of the Japanese credit dataset.

Expe. number/parameter	*k*	*η*	*M*	Testing accuracy
1	2	0.01	25	84.37 ± 2.16
2	2	0.03	28	85.39 ± 1.36
3	2	0.05	30	84.85 ± 1.61
4	2	0.07	32	85.39 ± 1.08
5	2.5	0.01	28	85.44 ± 1.40
6	2.5	0.03	25	85.29 ± 1.13
7	2.5	0.05	32	85.25 ± 1.52
8	2.5	0.07	30	85.54 ± 1.42
9	3	0.01	30	81.90 ± 8.84
10	3	0.03	32	85.01 ± 1.52
11	3	0.05	25	85.08 ± 1.51
12	3	0.07	28	84.90 ± 1.37
13	3.5	0.01	32	82.12 ± 7.96
14	3.5	0.03	30	84.79 ± 1.05
15	3.5	0.05	28	83.42 ± 5.14
16	3.5	0.07	25	83.94 ± 2.42

**Table 6:** Structures of PNN and MLP for the Australian and Japanese credit datasets.

Dataset	Model	Number of input	Number of branch hidden node	Number of output	Number of adjusted parameters
Australian	PNN	14	30	1	840
	MLP	14	53	1	849
Japanese	PNN	15	30	1	900
	MLP	15	53	1	902

**Table 7:** Comparison of the simulation results between PNN and MLP of the Australian credit dataset.

Method	Accuracy (%)	*p* value	Sensitivity	Specificity	AUC
PNN	85.64 ± 1.74	N/A	0.9484	0.9111	0.9411
MLP	84.23 ± 1.73	0.0038	0.9063	0.7789	0.8976

**Table 8:** Classification accuracy rates comparison between PNN and other algorithms obtained from literatures of the Australian credit dataset.

Authors (published year)	Algorithms (train-to-test ratios)	Classification accuracy rate (%)
Luo et al. (2009) [48]	SVM (10 × CV)	80.43

Peng et al. (2011) [49]	Bayesian network (10 × CV)	85.22
	KNN (10 × CV)	79.42
	RBF network (10 × CV)	83.04

Yu et al. (2011) [1]	C4.5 (10 × CV)	84.3
	LVQ (10 × CV)	82.97

Chang and Yeh (2012) [50]	SVM (10 × CV)	84.7
	C4.5 (10 × CV)	82.5
	Naive Bayes (10 × CV)	84.9

Zhu et al. (2013) [51]	QDA (5 × CV)	80.02
	DT (5 × CV)	83.18

Tsai et al. (2014) [52]	MLP (10 × CV)	82.44
	DT (10 × CV)	84.91

Lessmann et al. (2015) [8]	CART (10 × CV)	66.4
	ELM (10 × CV)	69.8
	LDA (10 × CV)	78.9
	Logistic regression (10 × CV)	80.7
	ADT (10 × CV)	79.8
	Bag (10 × CV)	76.8
	Boost (10 × CV)	81
	Random forest (10 × CV)	85.2

Khashei and Mirahmadi (2015) [7]	QDA (50%-50%)	80.1
	SVM (50%-50%)	77.5

Our method (2017)	PNN (50%-50%)	85.64
	PNN (5 × CV)	85.31
	PNN (10 × CV)	85.19

**Table 9:** Comparison of the simulation results between PNN and MLP of the Japanese credit dataset.

Method	Accuracy (%)	*p* value	Sensitivity	Specificity	AUC
PNN	85.54 ± 1.42	N/A	0.9333	0.8316	0.9301
MLP	83.92 ± 1.32	6.4811*e*^−05^	0.8247	0.8148	0.8900

**Table 10:** Classification accuracy rates comparison between PNN and other algorithms obtained from the literatures of the Japanese credit dataset.

Authors (published year)	Algorithms (train-to-test ratios)	Classification accuracy rate (%)
Yu et al. (2008) [53]	log⁡*R* (10 × CV)	75.82
	ANN (10 × CV)	80.77
	SVM (10 × CV)	79.91
	Neuro-fuzzy hybrid (10 × CV)	77.91
	Fuzzy SVM hybrid (10 × CV)	83.94

Tsai et al. (2014) [52]	MLP (10 × CV)	84.38

Our method (2017)	PNN (50%-50%)	85.54
	PNN (5 × CV)	85.23
	PNN (10 × CV)	85.27

**Table 11:** Model structure comparison between Australian and Japanese credit datasets.

Dataset	Feature input		Dendritic layer		Adjusted weight	
	Initial value	Selected value	Initial value	Selected value	Initial value	Selected value
Australian credit dataset	14	4	30	2	840	16
Japanese credit dataset	15	4	30	2	900	16

**Table 12:** Verification between LC and CP.

Credit dataset	Test accuracy of PNN (%)	Test accuracy of LC (%)	Test accuracy of CP (%)
Australian	85.64	85.80	61.21
Japanese	85.54	85.51	65.56

**Table 13:** Extraction rate and accuracy rate comparison between PNN and other feature selection methods of the Australian credit dataset.

Methods	Numbers of variables before feature selection	Numbers of variables after feature selection	Extraction rate (%)	Accuracy rate (%)
*T*-test [54]	14	12	14.3	89.27
Stepwise [54]	14	7	50	84.74
Related matrix [54]	14	12	14.3	89.31
Factor analysis [54]	14	9	35.7	86.08
PCA [54]	14	9	35.7	89.93
PNN	14	4	71.4	85.80

**Table 14:** Extraction rate and accuracy rate comparison between PNN and other feature selection methods of the Japanese credit dataset.

Methods	Numbers of variables before feature selection	Numbers of variables after feature selection	Extraction rate (%)	Accuracy rate (%)
*T*-test [54]	15	12	20	65.53
Stepwise [54]	15	5	66.7	82.64
Related matrix [54]	15	12	20	60.16
Factor analysis [54]	15	11	26.7	74.22
PCA [54]	15	8	46.7	74.00
PNN	15	4	73.3	85.51

## References

[1] Yu L., Yao X., Wang S., Lai K. K. (2011). Credit risk evaluation using a weighted least squares SVM classifier with design of experiment for parameter selection. *Expert Systems with Applications*.

[2] Lee T.-S., Chiu C.-C., Lu C.-J., Chen I.-F. (2002). Credit scoring using the hybrid neural discriminant technique. *Expert Systems with Applications*.

[3] West D. (2000). Neural network credit scoring models. *Computers & Operations Research*.

[4] Thomas L. C., Edelman D. B., Crook J. N. (2002). *Credit scoring and its applications*.

[5] Lin W.-Y., Hu Y.-H., Tsai C.-F. (2012). Machine learning in financial crisis prediction: A survey. *IEEE Transactions on Systems, Man, and Cybernetics, Part C: Applications and Reviews*.

[6] Abdou H. A., Pointon J. (2011). Credit scoring, statistical techniques and evaluation criteria: a review of the literature. *Intelligent Systems in Accounting, Finance and Management*.

[7] Khashei M., Mirahmadi A. (2015). A soft intelligent risk evaluation model for credit scoring classification. *International Journal of Financial Studies*.

[8] Lessmann S., Baesens B., Seow H.-V., Thomas L. C. (2015). Benchmarking state-of-the-art classification algorithms for credit scoring: An update of research. *European Journal of Operational Research*.

[9] Hand D. J., Henley W. E. (1997). Statistical classification methods in consumer credit scoring: A review. *Journal of the Royal Statistical Society. Series A: Statistics in Society*.

[10] Baesens B., Setiono R., Mues C., Vanthienen J. (2003). Using neural network rule extraction and decision tables for credit-risk evaluation. *Management Science*.

[11] Altman E. I., Marco G., Varetto F. (1994). Corporate distress diagnosis: comparisons using linear discriminant analysis and neural networks. *Journal of Banking & Finance*.

[12] Atiya A. F. (2001). Bankruptcy prediction for credit risk using neural networks: a survey and new results. *IEEE Transactions on Neural Networks and Learning Systems*.

[13] Carter C., Catlett J. (1987). Assessing Credit Card Applications Using Machine Learning. *IEEE Expert-Intelligent Systems and their Applications*.

[14] Leonard K. J. (1993). Detecting credit card fraud using expert systems. *Computers & Industrial Engineering*.

[15] Desai V. S., Conway D. G., Crook J. N., Overstreet G. A. (1997). Credit-scoring models in the credit-union environment using neural networks and genetic algorithms. *IMA Journal of Management Mathematics*.

[16] Varetto F. (1998). Genetic algorithms applications in the analysis of insolvency risk. *Journal of Banking & Finance*.

[17] Odom M. D., Sharda R. A neural network model for bankruptcy prediction.

[18] Klersey G., Dugan M. (1995). Substantial doubt: Using artificial neural networks to evaluate going concern. *Advances in accounting information systems 3*.

[19] McKee T. E., Greenstein M. (2000). Predicting bankruptcy using recursive partitioning and a realistically proportioned data set. *Journal of Forecasting*.

[20] Piramuthu S. (1999). Financial credit-risk evaluation with neural and neurofuzzy systems. *European Journal of Operational Research*.

[21] Koh H. C., Tan W. C., Peng G. C. (2004). Credit scoring using data mining techniques. *Singapore Management Review*.

[22] Bellotti T., Crook J. (2009). Support vector machines for credit scoring and discovery of significant features. *Expert Systems with Applications*.

[23] Trinkle B. S., Baldwin A. A. (2016). Research opportunities for neural networks: the case for credit. *Intelligent Systems in Accounting, Finance and Management*.

[24] Lim M. K., Sohn S. Y. (2007). Cluster-based dynamic scoring model. *Expert Systems with Applications*.

[25] Wang H., Xu Q., Zhou L. (2015). Large unbalanced credit scoring using lasso-logistic regression ensemble. *PLoS ONE*.

[26] Rosenblatt F. (1962). *Principles of Neurodynamics*.

[27] McCulloch W. S., Pitts W. (1943). A logical calculus of the ideas immanent in nervous activity. *Bulletin of Mathematical Biology*.

[28] Abbott L. F., Regehr W. G. (2004). Synaptic computation. *Nature*.

[29] Koch C., Poggio T., Torre V. (1983). Nonlinear interactions in a dendritic tree: Localization, timing, and role in information processing. *Proceedings of the National Acadamy of Sciences of the United States of America*.

[30] Blomfield S. (1974). Arithmetical operations performed by nerve cells. *Brain Research*.

[31] Brunel N., Hakim V., Richardson M. J. E. (2014). Single neuron dynamics and computation. *Current Opinion in Neurobiology*.

[32] Torre V., Poggio T. (1978). A synaptic mechanism possibly underlying directional selectivity to motion. *Proceedings of the Royal Society B Biological Science*.

[33] Jan Y.-N., Jan L. Y. (2010). Branching out: Mechanisms of dendritic arborization. *Nature Reviews Neuroscience*.

[34] Wen Q., Chklovskii D. B. (2008). A cost-benefit analysis of neuronal morphology. *Journal of Neurophysiology*.

[35] Komatsu Y., Fujii K., Nakajima S., Umetani K., Toyama K. (1985). Electrophysiological and morphological correlates in the development of visual cortical circuitry in infant kittens. *Developmental Brain Research*.

[36] Koch C., Poggio T., Torre V. (1982). Retinal ganglion cells: a functional interpretation of dendritic morphology. *Philosophical Transactions of the Royal Society B: Biological Sciences*.

[37] Destexhe A., Marder E. (2004). Plasticity in single neuron and circuit computations. *Nature*.

[38] Koch C. (1997). Computation and the single neuron. *Nature*.

[39] Reynolds J. N. J., Wickens J. R. (2002). Dopamine-dependent plasticity of corticostriatal synapses. *Neural Networks*.

[40] Gu Q. (2003). Contribution of acetylcholine to visual cortex plasticity. *Neurobiology of Learning and Memory*.

[41] Losonczy A., Makara J. K., Magee J. C. (2008). Compartmentalized dendritic plasticity and input feature storage in neurons. *Nature*.

[42] Chen W., Sun J., Gao S., Cheng J.-J., Wang J., Todo Y. (2017). Using a single dendritic neuron to forecast tourist arrivals to Japan. *IEICE Transaction on Information and Systems*.

[43] Yu Y., Wang Y., Gao S., Tang Z. (2017). Statistical modeling and prediction for tourism economy using dendritic neural network. *Computational Intelligence and Neuroscience*.

[44] Jiang T., Gao S., Wang D., Ji J., Todo Y., Tang Z. (2017). A neuron model with synaptic nonlinearities in a dendritic tree for liver disorders. *IEEJ Transactions on Electrical and Electronic Engineering*.

[45] Ji J., Gao S., Cheng J., Tang Z., Todo Y. (2016). An approximate logic neuron model with a dendritic structure. *Neurocomputing*.

[46] Tang Z., Tamura H., Kuratu M., Ishizuka O., Tanno K. (2001). A model of the neuron based on dendrite mechanisms. *Electronics and Communications in Japan, Part III: Fundamental Electronic Science (English translation of Denshi Tsushin Gakkai Ronbunshi)*.

[47] Paolicelli R. C., Bolasco G., Pagani F. (2011). Synaptic pruning by microglia is necessary for normal brain development. *Science*.

[48] Luo S.-T., Cheng B.-W., Hsieh C.-H. (2009). Prediction model building with clustering-launched classification and support vector machines in credit scoring. *Expert Systems with Applications*.

[49] Peng Y., Wang G., Kou G., Shi Y. (2011). An empirical study of classification algorithm evaluation for financial risk prediction. *Applied Soft Computing*.

[50] Chang S.-Y., Yeh T.-Y. (2012). An artificial immune classifier for credit scoring analysis. *Applied Soft Computing*.

[51] Zhu X., Li J., Wu D., Wang H., Liang C. (2013). Balancing accuracy, complexity and interpretability in consumer credit decision making: A C-TOPSIS classification approach. *Knowledge-Based Systems*.

[52] Tsai C.-F., Hsu Y.-F., Yen D. C. (2014). A comparative study of classifier ensembles for bankruptcy prediction. *Applied Soft Computing*.

[53] Yu L., Wang S., Lai K. K. (2008). Credit risk assessment with a multistage neural network ensemble learning approach. *Expert Systems with Applications*.

[54] Tsai C.-F. (2009). Feature selection in bankruptcy prediction. *Knowledge-Based Systems*.

[55] Koch C. (2004). *Biophysics of Computation: Information Processing in Single Neurons*.

[56] Sietsma J., Dow R. J. Neural net pruning-why and how.

[57] Asuncion A., Newman D. J. http://www.ics.uci.edu/mlearn/MLRepository.

[58] Wang C.-M., Huang Y.-F. (2009). Evolutionary-based feature selection approaches with new criteria for data mining: A case study of credit approval data. *Expert Systems with Applications*.

[59] Taguchi G., Jugulum R., Taguchi S. (2004). *Computer-Based Robust Engineering: Essentials for DFSS*.

[60] Khashman A. (2011). Credit risk evaluation using neural networks: emotional versus conventional models. *Applied Soft Computing*.

[61] Coussement K., Buckinx W. (2011). A probability-mapping algorithm for calibrating the posterior probabilities: A direct marketing application. *European Journal of Operational Research*.

[62] Haykin S. S. (2009). *Neural Networks and Learning Machines*.

[63] Yang J., Olafsson S. (2006). Optimization-based feature selection with adaptive instance sampling. *Computers & Operations Research*.

